# Metagenome-assembled genome sequence of *Spiroplasma phoeniceum*, assembled from the hindgut of *Locusta migratoria*, a migration grasshopper species

**DOI:** 10.1128/mra.00353-24

**Published:** 2024-07-16

**Authors:** Jaeha Kim, Takumi Murakami, Atsushi Toyoda, Hiroshi Mori

**Affiliations:** 1 Genome Diversity Laboratory, National Institute of Genetics, Mishima-shi, Japan; 2 School of Life Science and Technology, Tokyo Institute of Technology, Oookayama, Japan; 3 Advanced Genomics Center, National Institute of Genetics, Mishima-shi, Japan; Montana State University, Bozeman, Montana, USA

**Keywords:** metagenomics, genome analysis

## Abstract

*Spiroplasma phoeniceum* is a plant pathogen and a mesophilic microaerophile. Here, we report the metagenome-assembled genome (MAG) sequence of *S. phoeniceum* binned from hindgut contents of the wild-type male *Locusta migratoria*, a grasshopper species. The MAG sequence comprises 1,059,205 bp in 91 contigs with a 26.3% of GC content.

## ANNOUNCEMENT

*Spiroplasma phoeniceum* is a plant pathogen and mesophilic microaerophile. It was isolated in culture from plants of *Catharanthus roseus* in Syria in 1987 and was reported as *S. phoeniceum* P40 (1). The complete genome of *S. phoeniceum* P40 was reported in 2019 (2). Here, we introduce a metagenome-assembled genome (MAG) sequence of *Spiroplasma phoeniceum* binned from hindgut contents of the wild-type male *Locusta migratoria*, a grasshopper species.

A wild-type male *L. migratoria* was gathered from Kisegawa area (lat. 35.10325, long. 138.88771) in Japan in July 2022 and immediately was moved to a −30°C refrigerator. After that, the hindgut of *L. migratoria* was carefully extracted by the dissection on ice. For the DNA isolation, QIAamp Fast DNA Stool Mini Kit (Qiagen GmbH, Hilden, Germany) was used based on the standard DNA isolation protocol. For the DNA quantification, Qubit (Life Technologies, CA, USA.) was used. The quality of the DNA-eluted solution was checked with a Bioanalyzer (Agilent 2100 Bioanalyzer, Agilent Technologies, CA, USA.). Sequencing libraries were generated using the Illumina Nextera Flex Kit (Illumina, San Diego, CA, USA.). The libraries were sequenced on the Illumina NovaSeq 6000 platform, and 150-bp paired-end reads were generated. As a result, 33,715,814 read pairs were obtained. The read quality filtering (i.e., adaptor sequence removal and low-quality sequence trimming) was performed using fastp v.0.23.2 (3). For removing host DNA sequences, *L. migratoria* genome sequence (NCBI GenBank ID: GCA_026315105.1) was referred to as the reference for BWA-MEM v.0.7.17 (4) read mapping with the default parameters. MEGAHIT v.1.2.9 (5) was used for the assembly from reads to contigs with the default parameters. Bacterial contig sequences were identified, and unassigned sequences were excluded using Kraken2 v.2.0.8 (6) based on the standard Kraken 2 databased v.Sep.2022. For the contig binning, the read mapping result of BWA-MEM v.0.7.17 (4) against bacterial contigs was used. Metagenome-assembled genomes were constructed by using MetaWRAP v.1.3.2 (7). The taxonomic name was inferred by GTDB-Tk v.2.1.1 (8). The MAG was reassembled by mapping to the reference genome *S. phoeniceum* P40 (GCF_003339775.1) using RagTag v.2.1.0 (9). Protein-coding genes on the MAG were annotated using DFAST v.1.2.0 (10). To compare genomes with the same annotation methods, genome sequences of *S. phoeniceum* P40 (GCF_003339775.1) and *S. phoeniceum* MAG PmF2021_Spiro (CP110829.1) and PmM2021_Spiro (CP110830.1) were obtained and reannotated using DFAST. The protein-coding genes of the four strains were clustered using OrthoFinder v.2.5.2 (11). Default parameters were used except where otherwise noted.

As a result, the 1,059,205-bp MAG (Lm2022) comprising 91 contigs (N50 = 834,464 bp) with 26.3% of GC content, 93.58% completeness, and 0.0% contamination was predicted. The average nucleotide identity (ANI) between *S. phoeniceum* P40 and Lm2022 is 96.52%, calculated using the OrthoANIu tool (12). The genome differences of four *S*. *phoeniceum* strains were described in Table 1. The MAG sequence provides important information about the genomic diversity of this species.

**TABLE 1:** The genome difference of four *S*. *phoeniceum* strains^*a*^ ^,*b*,*c*^

	Lm2022	P40	PmF2021_Spiro	PmM2021_Spiro
Genome ID	PRJDB17847	GCF_003339775.1	CP110829.1	CP110830.1
Genome size (bp)	1,059,205	1,908,276	2,171,854	2,179,570
No. of chromosomes or contigs	91	1	1	1
No. of plasmids	N.D.^*d*^	3	N.D.	N.D.
GC content (%)	26.3	25.4	24.4	24.4
No. of CDSs	1,106	2,619	3,040	3,052
No. of rRNA genes	1	3	3	3
No. of tRNA genes	31	43	31	31
No. of unique CDSs	145	1064	0	2

^*a*^ Lm2022: A MAG of *S. phoeniceum* isolated from hindgut of *L. migratoria*.

^*b*^ P40: The complete genome of *S. phoeniceum* P40 reported in 2019.

^*c*^ PmF2021_Spiro and PmM2021_Spiro: MAGs of *S. phoeniceum* reported in 2021.

^*d*^ N.D.,Not Detected.

## Data Availability

The metagenome assembled genome sequence data is available in the DDBJ under the accession number BAABXD010000001-BAABXD010000091. The BioProject number is PRJDB17847. The metagenomic sequencing raw read is deposited under DRA018389 with the BioSample numbers SAMD00765079 and SAMD00763153.
